# Investigating the role of E-commerce marketing capabilities to achieve the strategic performance of tourism firms

**DOI:** 10.3389/fpsyg.2023.1105539

**Published:** 2023-01-26

**Authors:** Jianchun Zhao, Peilin Zhang

**Affiliations:** Centre for Teaching Supervision and Evaluation, Shandong Management University, Jinan, Shandong, China

**Keywords:** China, customer engagement, E-commerce marketing capabilities, strategic performance, internet of things

## Abstract

**Introduction:**

This study aims to explore the relationships between strategic performance, e-commerce marketing capabilities (ECMCs), the Internet of Things (IoT) and customer engagement. This study examines the direct association between ECMC and strategic performance. Current research also explores the customer engagement mediation between ECMC and strategic performance (SP). Furthermore, our study investigates the IoT moderation between ECMC and SP.

**Methods:**

We test our research hypotheses using data collected in the tourism sector in the context of digital commerce. The questionnaire is used to collect data through random sampling, and these data are useful as a basis for future research. By adding e-commerce capabilities, we show firms how to become more efficient and improve their strategic performance. Moreover, this study, by incorporating the findings of the existing literature, provides a strong foundation for studying the impact of ECMCs and customer engagement on strategic performance as well as the mediating role of e-trust.

**Results:**

The results can be useful for managers who conduct digital business internationally, as they need to understand the importance of ECMCs. In fact, the adaptation of ECMCs to the organization enhances customer engagement and helps to improve strategic performance.

**Discussion:**

The approach used in this study is in line with previous theoretical analyses and shows emerging patterns in international digital businesses. Moreover, this study adds insights to the e-commerce research by linking different dimensions to reach an in-depth understanding of each item that is affected by ECMCs.

## 1. Introduction

Over the years, many researchers have tested theories about how marketing strategies can be used to engage customers to determine the differences between the developed and emerging market perspectives (Brodie et al., 2011). Traditional business techniques are rapidly evolving into electronic marketing and thus businesses need mechanisms to adopt these changes and achieve strategic performance (SP; Chakravarthy, 1986). In this scenario, e-commerce marketing capabilities (ECMCs) rapidly adopt such changes and foster customer engagement to support strategic performance, which is implemented through digital technologies (Palmu, 2011). ECMCs are linked to customer engagement and influence SP. ECMCs are described as the capability of a company to recognize, improve, and integrate e-commerce tactics into its product offerings to reach selected markets (Ardiansah et al., 2021, June). Because ECMCs involve a specific marketing capability, they are seen by several authors as being one of the processes that aid organizations in realizing their strategies (Yousaf and Majid, 2018). However, there is a need to explore how customer engagement can be enhanced through ECMCs in the digital age (Lane, 2009). In fact, firms can benefit from e-commerce technologies by engaging their customers and becoming more connected with other businesses (Czinkota et al., 2021).

Many other advantages can be gained by embracing e-commerce, such as an improved ability to personalize sales techniques, e.g., simplified electronic auditing, simplified information exchange with key stakeholders and order entry processing, shipment tracing, enhanced relations with existing clients, and better consumer awareness of products/services (Ubags et al., 2021). Such benefits demonstrate that there is a need for organizations to evolve from using traditional methods by embracing advanced techniques and leveraging ECMCs, as they help customers to achieve their goals through e-commerce capabilities and can, at the same time, enhance firms’ strategic performance (Ittner et al., 2003). Moreover, it is also important for practitioners and scholars to take into account the significant role played by customer engagement. This topic is a key driver of improving firms’ strategic performance through e-commerce marketing capabilities (Verhoef et al., 2010).

Due to the development of new digital technologies, customers are engaged in different ECMC e-business processes by liking, sharing and commenting on them (Micheli and Manzoni, 2010). Thus, this study analyzes the roles of ECMCs and customer engagement on firms’ strategic performance. In this respect, this paper sheds light on the fact that ECMCs increase customer engagement, which can improve service quality as well as strategic performance. Moreover, ECMCs bring about advancements in buying and selling processes while collecting information about the needs and expectations of consumers, which is key for improving strategic performance (Firoozi et al., 2020). Previous studies highlighted numerous outcomes of the use of ECMCs in business, e.g., performance (e.g., Gregory et al., 2019), management competency, and operations (Ramanathan et al., 2012); although its most important outcome—its effect on strategic performance—has not been fully investigated.

In fact, ECMCs have brought about changes in businesses globally and yield desirable benefits through increased customer engagement and earnings as well as reduced costs (Bowden, 2009). Tourism firms are bestowed with hydro and solar energy potential that, if exploited in a systematic manner, can ensure their energy security over time. The revolution of the traditional marketplace into e-marketing particularly requires e-commerce capabilities in a firm toward improving firms’ strategic performance (Atkinson et al., 1997; Fukuyama and Tan, 2022). ECMCs provide numerous opportunities to improve communication among distributors and buyers, purchasers, and suppliers. Recently, e-commerce capabilities have become an essential practice for engaging customers (Rahimi, 2021). The rapid development in e-commerce allows organizations to leverage new and specialized capabilities to achieve successful customer engagement (Kaplan, 2001). It is important for firms to implement unique e-commerce marketing capabilities to attain optimal strategic performance. Though, to the best of our knowledge, merely few studies have been carried out that examine developing capabilities such as e-commerce (Gregory et al., 2019), logistic capability (Cho et al., 2008) and seller marketing capability (Mu and Zhang, 2021) etc. but there remains a gap regarding the different mediation and moderation factors for enhancing and increasing strategic performance. However, no research has been conducted that mutually examine the impact of e-commerce marketing capabilities, customer engagement and IoT on strategic performance. Hence, to fill up this literature gap our study aims to examine the direct affect of ECMC through mediation of the customer engagement, and moderating role of IoT by choosing tourism firms as a case study. Exploring of all selected variables influence can support us in improved understanding of how ECMC influence strategic performance of tourism firms.

**Figure 1:**
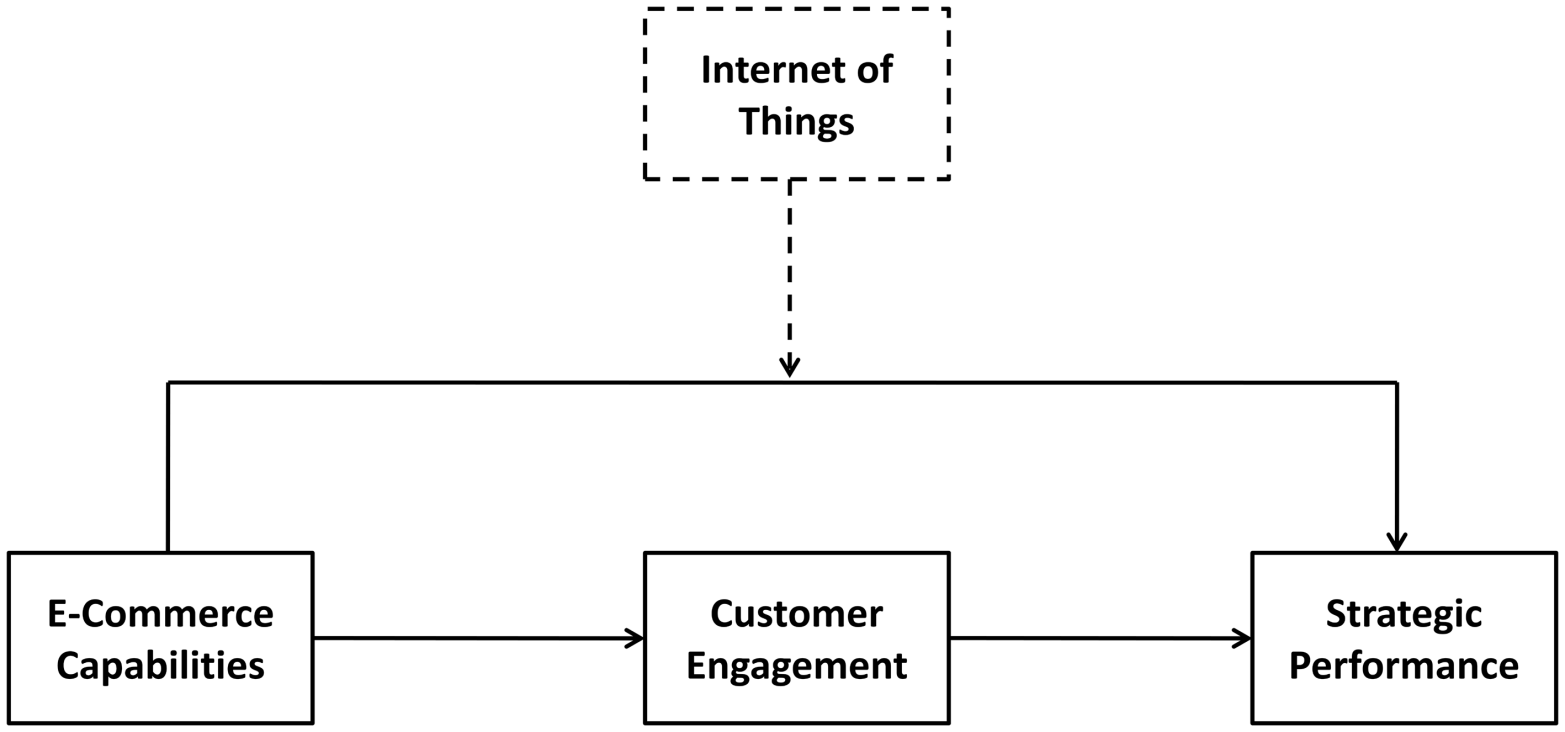
Theoretical framework.

To fill this research gap and guide managers in this field, our study expands upon the reasons behind effective strategic performance, including e-commerce marketing capabilities, the IoT and customer engagement, then evaluates the impact of these strategies on strategic performance (see Figure 1). This study extends the literature on the impact of ECMCs and customer engagement into the field of strategic performance. To address this gap, this study has primary three objectives

- First, how ECMC is positively associated with strategic performance?
- Second, does customer engagement mediates between ECMC and strategic performance?
- Third, do IoT perform moderation between ECMC and strategic performance?

The structure of this paper includes an introduction, a literature review, a presentation of our methodology and related findings, then a discussion and our conclusions. In the last section, the contributions and limitations of this study are summarized along with suggestions for future research. The next sections identify suitable approaches to and related research areas for analyzing the particular fields that will further develop the hypotheses of this study.

Virtually, psychological social support encourages in collaboration and support in apt emotional and physical activities to meet standards of the ethical consumption and natural environment *via* antecedent of the environmental knowledge. Nevertheless, this research model is unique and adds to previous literature information in these ways. Firstly, our study primary objective is to test that how environmental knowledge is directly associated with ethical consumption. Secondly, current study proposes to assess how CSR participation mediates in the linkage between environmental knowledge and ethical consumption. Thirdly, our study aims to investigate the level at which psychological social support can strengthen the linkage between environmental knowledge and ethical consumption. This research suggests some theoretical and practical bases or views for improvement of ethical consumption practices in tourism firms of Saudi Arabia. Current paper reminder is put as following; firstly, in section 2 we describe literature review of the selected study variables dependent on virtues of research constructs, secondly, in next section we explain methodology technique used in current research, in section 4 we include results and analysis and in last section we elucidate details of discussion and conclusion.

## 2. Literature review

ECMCs refers to an organization’s ability to use the Internet to facilitate transactions, share information, improve customer service, and build up back end integration (Gregory et al., 2019). ECMCs are the unique IT abilities that reflect any organization’s capability to adopt e-commerce capabilities to manage inter-and intra-organizational operations—for instance, collaboration between business partners, services rendered, and outside organizational relationships (Tsiotsou et al., 2010). Customer engagement refers to the ethical and emotional relationship between the customer, a company and its brand. Highly engaged customers promote and buy products and thus show more loyalty and trust to the company and its brand (Bolton, 2011). Customer engagement is a combination of different cognitive aspects, such as being interested in the activities of a company, the behavioral perspective (e.g., participating in company processes), and emotional aspects related to a positive feeling about a company’s activities (Vivek et al., 2012). CE refers toa customer’s emotional, cognitive and behavioral existence in a brand community through online websites (Bijmolt et al., 2010). The IoT refers to a network of interrelated physical objects—both digital and mechanical machines—that are equipped with software, processing ability, sensors and other technologies for the receiving and sharing of data (Khan and Salah, 2018). Strategic performance shows business performance and competitive position in significant areas such as industry awareness, penetration, and responsiveness (De Waal, 2013).

### 2.1. E-commerce marketing capabilities and strategic performance

ECMCs are considered the abilities that describe the firm’s initiatives and strategies for improving strategic performance (Zhu and Kraemer, 2002).ECMCs not only improve strategic performance, also have influence on business value which is measured through both financial and nonfinancial factors (Vasile et al., 2022). Organization can adapt to new environments by building, reconfiguring and integrating their external and internal competencies (Kotabe et al., 2008). Companies must rapidly adapt to new processes to fulfill the customers’ demands. Companies need e-commerce capabilities to speed up online transactions, customize their processes and enhance their strategic performance (Banker et al., 1996). IT capability, ECMCs, and strategic performance are difficult to separate. ECMCs allow customers to interact and participate in decision-making processes when buying product and services (Cassia and Magno, 2021). ECMCs enable firms to share information, develop user-demanded content, and interact socially with customers. They also allow firms to interact with customers to improve their strategic performance (Torres et al., 2014). Firm usage of ECMCs can improve strategic performance by increasing opportunities to interact with customers. ECMCs capture e-customer involvement, which is essential for the improvement of firm strategic performance (Cho et al., 2008). Currently, e-commerce capabilities have been an essential element in businesses growth. Some firms now invest in e-commerce to enhance their competitive advantages. E-commerce cannot improve financial performance alone but rather attains the targets of strategic performance (Saini and Johnson, 2005). Therefore, ECMCs have a directly positive impact on firms’ strategic performance.

*H1*: ECMCs are positively related with strategic performance.

### 2.2. Customer engagement as a mediator in the relationship between e-commerce marketing capabilities and strategic performance

Customer engagement should act as a mediator in the relationship between ECMCs and strategic performance. Customer engagement being an outcome of ECMCs is a critical mechanism in which customers can be capable of sharing and transforming their interests with firm management (Thakur, 2019). ECMCs enhance customers’ engagement, which positively impacts firm performance, as customers help them understand market demands. Enterprises with higher-level ECMCs are capable of building relationships with customers and responding to customers’ expectations (Ho et al., 2020). Hence, engaged customers help in improving strategic performance. This study proposed that customer engagement plays a mediating role in the relationship between ECMCs and strategic performance. Customers are the main foundation for introducing new products and services (Jermsittiparsert et al., 2018). ECMCs connect business with customers on a round-the-clock basis, which facilitates enterprises in creating new ideas and responding to customer needs (Ramanathan, 2010). Firms derive benefits from customers by launching ECMCs to engage and cooperate with them (Chenhall, 2005). Such customer engagement determines the level of strategic performance. Customer engagement is advantageous to brands by the information sharing and customer involvement it creates (Stone et al., 2019).

*H2*: Customer engagement mediates the relationship between ECMCs and strategic performance.

### 2.3. The IoT moderates the relationship between e-commerce marketing capabilities and customer engagement

Technological development adds to information exchange by means of connecting a wide range of machines and tools (Shafique et al., 2020).ECMCs are used for handling complaints from and sharing information with customers. At the same time, if businesses use IoT tools, the impact of ECMCs on customer engagement will be stronger (Lee and Lee, 2015). IoT applications have the ability to link customers and improve labor productivity, which plays a pivotal role in facilitating online transactions by reducing uncertainties (Reyna et al., 2018). The IoT is an essential component in businesses now because all businesses and customers are online (Wheelus and Zhu, 2020). When customers feel safe and protected, they provide their personal details without hesitating (Moliner et al., 2018). The IoT has a broad scope, which supports growth in the digital economy and brings about advanced opportunities, new abilities, and a new service model which relies upon communication among different devices all over the globe (Madakam et al., 2015). The IoT strengthens the relationship between ECMCs and customer engagement. Firms that use ECMCs have an increased awareness of customers and brands (Farida et al., 2017). The IoT increases ECMCs’ effects on customers’ willingness to engage in online buying and selling processes (Stiawan et al., 2017). Customers are also willing to contribute to firm activities when IoT applications are used (Stiawan et al., 2017). Finally, this study clarifies the moderating role of the IoT. Firms can leverage the optimistic impact of the IoT in the relationship between ECMCs and customer engagement. The IoT provides opportunities to increase firms’ knowledge, data flow and attractiveness to obtain informational advantages over their competitors (Fang, 2017). Market-related ECMCs allow organizations to anticipate changes and respond to consumers’ needs and expectations; thus, we expect that this association will be stronger if IoT applications are also used. In light of the above arguments, we suggest that the IoT plays a mediating role in the relationship between ECMCs and customer engagement.

*H3*: The IoT plays a positive mediating role in the relationship between ECMCs and customer engagement.

## 3. Methodology

This research is quantitative and random sampling method was used for the collection of data from different tourism firm. Data was collected through questionnaires with the help of the two research associates.

### 3.1. Data collection

Data were collected from nine registered tourism firms located in Xian, China. These firms are engaged in digital commerce and use e-commerce platforms as their functioning mechanism. They sell products and give services through online applications that are easily accessible to customers. Customers can purchase through their mobile phones using the Internet and official websites. Data were collected by sending questionnaires *via* email or post to the respondents/owners of different tourism firms. A letter is attached to the questionnaire that explains the objectives and implications of our research. Data collected by questionnaire consisted of 508 samples, of which 485 were use able for data analysis, giving a 95.4% return rate. Twenty-three responses were discarded due to incompleteness.

### 3.2. Measurement

The validity scale plays an imperative role in designing the survey tool. We used a pretested scale from previous experiential studies to ensure the reliability and validity of our research. E-commerce marketing capability refers to those capabilities that are connected to communication channels for providing online information to the business world. E-commerce marketing capabilities are the unique IT abilities that reflect any organization’s ability to manage inter-and intra-organizational operations and processes for instance, collaboration among business partners, services rendered to consumers, and outside relationships (Saini and Johnson, 2005). Independent variables related to e-commerce marketing capabilities were measured to determine how well firm achieve their strategic goals.

### 3.3. Measurement scale

#### 3.3.1. E-commerce marketing capabilities

ECMCs are used as the independent variable and measured through 15 items that were adapted from the work of Gregory et al. (2019). These items reflect how tourism firms achieve their e-commerce-related objectives through promotion of online products and services, online product catalogues, online ordering, online access, online payment, e-procurement, the e-marketplace, and e-fulfilment.

#### 3.3.2. Customer engagement

Customer engagement measurements were modified from a four-item scale adapted from the work of Puriwat and Tripopsakul, (2021). This construct measures the influence of different factors on customer engagement.

#### 3.3.3. Internet of things

For the measurement of Internet of Things, we adapted eight items from De Vass et al. (2018). The IoT asked about websites used by the firms. This variable asks about the critical role of the IoT in improving tourism performance.

#### 3.3.4. Strategic performance

Strategic performance is the dependent variable and measured using a four-item scale adapted from the work of Yousaf et al. (2018). This item evaluates how a firm has executed its marketing strategies such as distribution, merchandising, and promotion.

## 4. Analysis

### 4.1. Discriminant and construct validity

To check the association of the e-commerce marketing capabilities with strategic performance through the mediation of the customer engagement correlation and regression analysis was used in this study. To test the validity and reliability of the data, this study utilized discriminant and convergent validity and the results were satisfactory. Composite reliability (CR) was also satisfactory as it shows values greater than 0.50. Table 1 shows the results of the factor loading, alpha values, composite reliability and AVE.

**Table 1:** Validation and reliability.

Description	Item	FL	Cronbach’s alpha	CR	AVE
E commerce marketing capabilities	15	0.74–0.88	0.83	0.93	0.72
Customer engagement	04	0.76–0.85	0.86	0.94	0.76
Internet of things	09	0.72–0.86	0.84	0.96	0.74
Strategic performance	04	0.72–0.83	0.82	0.95	0.75

Table 2 describes the outcomes of the Model 1, Model 2, Model 3 and Model 4. Table 2 presents the CFA results and confirms the model fitness. Model 4 is the hypothesized model and the results were satisfactory (χ2 = 1056.21, df = 465; χ2/df = 2.271; RMSEA = 0.05; CFI = 0.94; GFI = 0.93).

**Table 2:** Confirmatory factor analysis (CFA).

Model description	χ2	Df	χ2/df	RMESA	GFI	CFI
4-factor model	1056.21	465	2.271	0.05	0.93	0.94
3-factor model	1144.57	340	3.366	0.13	0.85	0.86
2-factor model	1247.41	320	3.898	0.18	0.73	0.74
1-factor model	1367.21	355	3.851	0.22	0.65	0.66

### 4.2. Correlation

Table 3 presents the correlation results and the means, SDs and alphas of E-commerce marketing capabilities, customer engagement, IoT and strategic performance. E-commerce marketing capabilities are positively related and significant with strategic performance (*p* value = significant). Customer engagement mediates between ECMC and strategic performance (*p* value = 0.001). IoT moderates between ECMC and strategic performance (*p* value = 0.001). The VIF (variance inflation factors) scores corroborates that multi-collinearity is not an issue in current study as its values was less than 10.0.

**Table 3:** Mean, SD, and correlation matrix.

Variable	Mean	SD	Cronbach’s alpha	1	2	3	4	5	6	7	8
B. Age	2.97	0.15	0.85	1.00							
B. Size	1.34	0.22	0.83	0.116**	1.00						
Respondent experience	1.87	0.42	0.82	0.215**	0.86*	1.00					
Respondent education	1.62	0.46	0.84	−0.03	0.07	1.00	1.00				
E-commerce marketing capabilities	3.74	0.48	0.82	−0.02	−0.18	0.01	−0.10	1.00			
Customer engagement	3.43	0.36	0.86	0.04	−0.05	0.093*	−0.02	0.184**	1.00		
Internet of things	3.62	0.85	0.82	−0.09	−0.15	−0.04	0.042*	0.267**	0.367**	1.00	
Strategic performance	1.23	0.63	0.83	0.03	−0.12	−0.05	−0.12	0.340*	0.249**	0.385**	1.00

*p < 0.001, **p < 0.05.

### 4.3. Hypothesis testing

Table 4 shows the direct results of the hypothesis 1. H1 proposed that E-Commerce marketing capabilities positively influence on strategic performance. The results show that e-commerce marketing capability is one the major antecedents of strategic performance. This is intuitive because organizations can sustain their performance if these are available to customers on a round-the-clock basis. ECMCs are positively associated with SP (β value = 0.16**, *p* < 0.000), thus H1 is accepted.

**Table 4:** E-commerce marketing capabilities and strategic performance.

Model	Hypothesis description	Beta value	F	T Value	Sig	Remarks
Model #1	ECMC➔Strategic Performance	0.16	14.075	0.165	0.000	Accepted

Table 5 presents the mediating results of customer engagement. A mediation analysis was conducted with 5,000 bootstrap iterations at the 95% confidence interval. Table 5 also shows the outcomes of the indirect effect of E-commerce marketing capabilities on strategic performance through customer engagement (ECMC➔CE➔SP). The results of the indirect effect of e-commerce marketing capabilities on SP through customer engagement were significant and positive (ECMC➔CE➔SP). Customer engagement mediates the relationship between them (B = 0.144, Boot =0.124, Minimum = 0.1352, Maximum = 0. 2,686). Thus, H4 is also accepted.

**Table 5:** Mediating effect of customer engagement between ECMCs and strategic performance.

Path description			Beta	T-Value	SE	Sig
ECMCàCustomer Engagement (Path a)			0.3475	7.652	0.042	0.000
Customer EngagementàStrategic Performance (Path b)			0.2442	7.521	0.056	0.000
ECMCàStrategic Performance (Path c)			0.3265	3.652	0.032	0.000
ECMCàStrategic Performance (Path c’)			0.1834	1.421	0.064	0.127
*R^2^ = 0.1424;F = 36.7453; p = 0.000*						
Bootstrap for indirect effect of IV on DV through mediator “ab path”						
Model Detail	Data	Boot	SE	Lower	Upper	Sig
ECMC-CE-SP	0.144	0.124	0.34	0.1352	0.2686	0.000

H3 presents that IoT had stronger and positive influence on strategic performance when ECMC support was higher. Table 6 shows the moderating effect of the Internet of Things between ECMCs and customer engagement. Table 6 presents the results of IOT on the direct relationship between e-commerce marketing capabilities and customer engagement. The results (Beta = 0.31**) signify that the IoT is a robust and positive moderator that positively affects the relationship between ECMCs and customer engagement, and this effect is significant at the 1% level. Hence, it is confirmed that IoT strengthen the linkage between ECMC and SP.

**Table 6:** Moderating effect of the internet of things.

SP						
Detail	*β*	T	*β*	T	*β*	T Value
Step 1						
B. Age	0.10	0.13	0.11	1.11	0.12	0.12
B. Size	0.09	0.14	0.07	0.22	0.14	0.17
Respondent-education	0.13	0.16	0.14	0.23	1.08	1.22
Respondent-experience	0.11	0.16	0.15	0.34	0.07	0.05
Step 2						
ECMC	–	–	0.34*	8.86	0.38*	4.36
IOT	–	–	0.26*	6.364	0.34*	5.58
Step 3						
ECMC*IOT	–	–	–	–	0.31**	3.28
F-Statistics	–	7.19**	–	18.12*	–	16.46*
R2	–	0.05	–	0.26	–	0.27
R2	–	---	–	0.26	–	0.03

*p < 0.0001,**p < 0.05.

## 5. Discussion

The study concluded the capabilities that facilitate firms’ collaboration with their customers and highlight the mediating role of customer engagement in the relationship between e-commerce marketing capabilities and strategic performance. Our study supports three hypotheses and highlights important implications for both theory and practice. The first hypothesis shows that e-commerce marketing capabilities positively affect strategic performance. These findings are supported in a general analysis and confirm the findings of previous studies. The findings corroborate that ECMCs not only improve strategic performance but also have an influence on business value, which is measured through both financial and nonfinancial factors (Dushnitsky et al., 2022). ECMCs help organizations to adapt to new environments by building, reconfiguring and integrating their external and internal competencies (Kotabe et al., 2008). We hypothesize that advancement in e-commerce marketing capabilities allow organizations to engage customers and improve their strategic performance. Our H2 is supported by empirical evidence that shows that customer engagement plays a mediating role in the relationship between ECMCs and strategic performance. Customer engagement plays an important role in achieving strategic performance among all firms. Few studies virtually investigate the effect of ECMCs on strategic performance *via* customer engagement (Yousaf and Majid, 2016). This customer engagement helps firms obtain knowledge of and information about opportunities to overcome achieve high strategic performance.

Customers are the main foundation for introducing new products and services (Jermsittiparsert et al., 2018). ECMCs connect business with customers on a round-the-clock basis, and such customer involvement facilitates enterprise in creating new ideas and responding to customer needs (Ramanathan, 2010). Firms derive benefits from customers by launching ECMCs to engage and cooperate with them (Chenhall, 2005). Customer engagement gives brands value and competitive advantages by sharing information and involving their customers (Stone et al., 2019). The findings support the argument that customer engagement is a key factor in achieving strategic performance. H3 in this study considered the mediating role of the IoT in the relationship between e-commerce marketing capabilities and customer engagement. Organizations attract customers through e-commerce capabilities when IoT applications are employed and customers act as a source of information. The results corroborate the view that the IoT is an essential component in businesses now because all businesses are online and it plays a strong role in the relationship between customers and the firm (Wheelus and Zhu, 2020). When customers feel safe and protected, they are more willing to provide their personal details (Moliner et al., 2018). The IoT has broad scope, which boosts growth in the digital economy and brings about advanced opportunities, new abilities, architectures and a new service model that facilitates communication among devices all over the globe (Madakam et al., 2015). The IoT strengthens the relationship between ECMCs and customer engagement to form independent hierarchical structures. The IoT strengthens ECMCs and customer relations by engaging customers in mutual dialogue.

### 5.1. Implications

In conclusion, this article presents a framework which clarifies and explains how ECMCs impact strategic performance. Considering that framework, ECMCs act as foundation for conventional customer engagement, which enhances strategic performance. Thus, we suggest that firm management should focus on ECMC of their employees and provide training sessions to them for improving its strategic performance. Customer engagement mediates the relationship between ECMCs and improved strategic performance. Hence, this study recommends that firm can increase customer engagement through offering good packages and improving strategic performance. Therefore, e-commerce capabilities enable customer engagement by providing them with knowledge and information and using their ECMC for higher outcomes. In addition, this study makes a novel contribution to the literature on ECMC, customer engagement and strategic performance. As an emerging technology, customers play an active role in buying services and products through the IoT. This implies that organizations are capable of effective customer engagement. The empirical results show that creating IoT capability enables organizations to build a platform for customers that guide toward strategic performance. The theoretical implications of H1 are that ECMCs are available to all organizations for enhancing their strategic performance. The findings support the view that customer engagement directly affects firms’ strategic performance. Customers are the main foundation for firms adopting new technologies and improving their strategic performance. Moreover, customer engagement mediates the relationship between ECMCs and strategic performance. Furthermore, the main practical implication of our study is that ECMCs can be expanded to create platforms through which customers are capable of modifying their experiences. The adaptation of ECMCs enhances customer engagement and helps to improve strategic performance.

### 5.2. Limitations and future directions

It must be highlighted that this research has several limitations although it gives insights for further research. The analysis of the relationships among concepts was developed at a single point in time, which means that it may be interesting to create longitudinal studies that can strengthen the exploration of this field of study and help to better understand the effects of the selected items in different time periods. Second, this article was built on data collected in China. Thus, future papers can take different geographic into account regions and compare their results with our findings to better explore the importance of ECMCs and to make our results more generalizable. Third, current study is conducted on tourism firms; in future researcher should conduct research on some other sectors such as SMEs, agricultural firms and industries. Finally, this study used customer engagement as a mediator and IoT as a moderator; other studies in future might use other mediating variables in this empirical model. To test its impacts on other regions and country.

## Data availability statement

The raw data supporting the conclusions of this article will be made available by the authors, without undue reservation.

## Ethics statement

Ethical review and approval was not required for the study on human participants in accordance with the local legislation and institutional requirements. Written informed consent from the patients/participants or patients/participants legal guardian/next of kin was not required to participate in this study in accordance with the national legislation and the institutional requirements.

## Author contributions

JZ wrote the manuscript and was responsible for funding matters. PZ collected the data and designed the methodology and estimation. All authors contributed to the article and approved the submitted version.

## Funding

The paper is sponsored by the Research on the construction of Teaching Quality Assurance System in application-oriented universities based on PDCA cycle model. Project number: 2021ZC042.

## Conflict of interest

The authors declare that the research was conducted in the absence of any commercial or financial relationships that could be construed as a potential conflict of interest.

## Publisher’s note

All claims expressed in this article are solely those of the authors and do not necessarily represent those of their affiliated organizations, or those of the publisher, the editors and the reviewers. Any product that may be evaluated in this article, or claim that may be made by its manufacturer, is not guaranteed or endorsed by the publisher.
